# Supplementary material for: Fetal outcomes and associated factors of antepartum hemorrhage in Ethiopia: A systematic review and meta-analysis
Source: PLoS One. 2025 Mar 4;20(3):e0319512. doi: 10.1371/journal.pone.0319512 (PMC11878924; doi:10.1371/journal.pone.0319512)
Supplement: S2 Table — (DOCX) [file pone.0319512.s002.docx]

**S2 Table: search strategy**

| **Database** | **keywords** | **Date** | **Results** |
| --- | --- | --- | --- |
| Pubmed | (("assessment," "prevalence," "magnitude," "associated factors," "determinants," "antepartum hemorrhage," "placenta previa," "placental abruption," "pregnant women") AND ("antepartum hemorrhage" OR "placenta previa" OR "placental abruption" OR "fetal outcomes and associated factors of antepartum hemorrhage among women in Ethiopia")). | February 2024 | 20 |
| Google Schoolar | TITLE-APH-KEY ((("assessment," "prevalence," "magnitude," "associated factors," "determinants," "antepartum hemorrhage," "placenta previa," "placental abruption," "pregnant women") AND ("antepartum hemorrhage" OR "placenta previa" OR "placental abruption" OR "fetal outcomes and associated factors of antepartum hemorrhage among women in Ethiopia"))). | March 2024 | 200 |
| Web Science | Find articles with these terms (("assessment," "prevalence," "magnitude," "associated factors," "determinants," "antepartum hemorrhage," "placenta previa," "placental abruption," "pregnant women") AND ("antepartum hemorrhage" OR "placenta previa" OR "placental abruption" OR "fetal outcomes and associated factors of antepartum hemorrhage among women in Ethiopia")). | April 2024 | 5 |
| Google | Boolean operator (("prevalence," "magnitude," "fetal outcome," "feto-maternal outcome," "associated factors," "determinants," "antepartum hemorrhage," "placenta previa," and "placental abruption")) AND ("antepartum hemorrhage" OR "placenta previa" OR "placental abruption" OR "fetal outcomes and associated factors of antepartum hemorrhage among women in Ethiopia")). | May 2024 | 300 |
