# Supplementary material for: Fetal outcomes and associated factors of antepartum hemorrhage in Ethiopia: A systematic review and meta-analysis
Source: PLoS One. 2025 Mar 4;20(3):e0319512. doi: 10.1371/journal.pone.0319512 (PMC11878924; doi:10.1371/journal.pone.0319512)
Supplement: S3 Table — (DOCX) [file pone.0319512.s003.docx]

Following the initial filtering process, we performed an in-depth review of the full texts of 25 publications to thoroughly evaluate their relevance and quality. After applying our stringent inclusion criteria, six studies were selected for inclusion in our systematic review and meta-analysis, ensuring a focused and comprehensive synthesis of the available evidence.

The studies included in the review, as well as those excluded, are summarized in the tables below. The excluded studies are accompanied by the specific reasons for their exclusion.

**S3 Table: Studies Included in the Systematic Review and Meta-Analysis**

| **Study** | | **Inclusion** | **Data Extractor** | **Date** |
| --- | --- | --- | --- | --- |
|  | Hailu H, Fisseha G, Goba G, Teka H, Ahmed S, Legesse AY. Incidence, Associated Factors and Outcomes of Antepartum Hemorrhage At Ayder Comprehensive Specialized Hospital and Mekelle General Hospital, Mekelle, Tigray, Ethiopia. Ethiop J Reprod Heal. 2023;15. | Included | Alex Ayenew | March 2024 |
|  | Chufamo N, Segni H, Alemayehu YK. Incidence, Contributing Factors and Outcomes of Antepartum Hemorrhage in Jimma University Specialized Hospital, Southwest Ethiopia. Univers J Public Heal. 2015;3: 153–159. doi:10.13189/ujph.2015.030403 | Included | Fikadu Wake | March 2024 |
|  | Kassie D. Management Outcome and Associated Factors Among Women With Antepartum Hemorrhage in Mettu Karl Referral Hospital , Oromia Regional State , Illu Ababora Zone , South West Ethiopia. 2019; 1–45. Available: http://10.140.5.162//handle/123456789/4081. | Included | Gemeda Wakgari | February 2024 |
|  | Assefa A, Fantahun Y, Mesfin E. Maternal and Perinatal Outcome of Antepartum Hemorrhage At Three Teaching Hospitals in Addis Ababa, Ethiopia. Ethiop J Reprod Heal. 2020;12: 12–19. | Included | Fikadu Wake | March 2024 |
|  | Gelan M, Bekela T, Angasu K, Ebisa M. Adverse Perinatal and Maternal Outcomes and Associated Factors among Women with Antepartum Hemorrhage in Jimma University Medical Center, Southwest Ethiopia, 2020. Obstet Gynecol Int. 2022;2022. doi:10.1155/2022/4594136 | Included | Alex Ayenew | May 2024 |
|  | Zegeye AM, Bazezew Y, Adare A, Jaleta P, Kumlachew W, Liben SW, et al. Determinants of feto-maternal outcomes of antepartum hemorrhage among women who gave birth in Awi zone public hospitals, Ethiopia. A case-control study. PLoS One. 2024;19: 1–16. doi:10.1371/journal.pone.0297700 | Included | Fikadu Wake | May 2024 |
