# Supplementary material for: Fetal outcomes and associated factors of antepartum hemorrhage in Ethiopia: A systematic review and meta-analysis
Source: PLoS One. 2025 Mar 4;20(3):e0319512. doi: 10.1371/journal.pone.0319512 (PMC11878924; doi:10.1371/journal.pone.0319512)
Supplement: S4 Table — (DOCX) [file pone.0319512.s004.docx]

**S4 Table: Reasons for Exclusion of Studies Identified During the Literature Search**

| **Study** | | **Causes of exclusion** | | **Data Extractor** | **Date** |
| --- | --- | --- | --- | --- | --- |
|  | Tadese M, Dagne K, Wubetu AD, Abeway S, Bekele A, Misganaw Kebede W, Baye Mulu G. Assessment of the  adverse pregnancy outcomes and its associated factors among deliveries at Debre Berhan Comprehensive Specialized Hospital, Northeast Ethiopia. Plos one. 2022 Jul 8;17(7):e0271287. | | Different outcome | Alex Ayenew | March 2024 |
|  | Dibaba B, Edosa D, Hajure M, Gebre G. Risk Factors of Antepartum Hemorrhage Among Mothers Who Gave Birth at Suhul General Hospital, 2016: A Case–Control Study. Journal of Multidisciplinary Healthcare. 2021 Feb 4:271-8. | | Different outcome | Adamu Shibabaw | May 2024 |
|  | Abdo RA, Halil HM, Birhanu LZ, Defara DA, Kebede BA. Prevalence and Associated Factors of Adverse Birth  Outcome Among Deliveries at Butajira Hospital,Southern Ethiopia. J Gynecol Womens Health. 2019;15(4):555920. https://doi.org/10.19080/JGWH.2019.15.555920. | | Different outcome | Gemeda Wakgari | February 25, 2024 |
|  | Cherie N, Mebratu A. Adverse birth out comes and associated factors among delivered mothers in Dessie referral  hospital, north East Ethiopia. J Womens Health Reprod Med. 2017;1(1):4 | | Different outcome | Fikadu Wake | March 2024 |
|  | Kassahun EA, Mitku HD, Getu MA. Adverse birth outcomes and its associated factors among women who delivered in north Wollo zone, Northeast Ethiopia: a facility based cross-sectional study. BMC Res Notes. 2019;12(1):357. | | Different outcome | Gemeda Wakgari | February 25, 2024 |
|  | Mihretie GN, Habitamu A. Pregnancy outcomes among women who gave birth at health institutions: A cross‐sectional study. Health Science Reports. 2022 Sep;5(5):e843. | | Different outcome | Alex Ayenew | March 2024 |
|  | Workie H. Adverse neonatal outcomes and associated risk factors in public and private hospitals of Mekelle city,  Tigray, Ethiopia: Unmatched case-control study. Neonat Pediatr Med. 2018;04(8). | | Not cross sectional | Adamu Shibabaw | May 2024 |
|  | Kebede E, M KekulawalaRisk factors for stillbirth and early neonatal death: a case-control study in tertiary hospitals in Addis Ababa, Ethiopia https://link.springer.com/article/10.1186/s12884-021-04025-8 | | Full text not accesible | Fikadu Wake | March 2024 |
|  | Workie H. Adverse neonatal outcomes and associated risk factors in public and private hospitals of Mekelle city,  Tigray, Ethiopia: Unmatched case-control study. Neonat Pediatr Med. 2018;04(8). | | Unclear data | Gemeda Wakgari | February 26, 2024 |
|  | Eshete A, Birhanu D, Wassie B. Birth outcomes among laboring mothers in selected health facilities of north  Wollo zone, Northeast Ethiopia: A facility based cross-sectional study. Health. 2013;5:1141–50 | | Different outcome | Alex Ayenew | March 2024 |
|  | Astatikie G, Limenih MA, Kebede M. Maternal and fetal outcomes of uterine rupture and factors associated  with maternal death secondary to uterine rupture. BMC pregnancy and childbirth. 2017 Dec;17:1-9. | | Different outcome | Adamu Shibabaw | May 2024 |
|  | Seyom E, Abera M, Tesfaye M, Fentahun N. Maternal and fetal outcome of pregnancy related hypertension in  Mettu Karl Referral Hospital, Ethiopia. Journal of ovarian research. 2015 Dec;8:1-7. | | Different outcome | Gemeda Wakgari | February 26, 2024 |
|  | Kassa GM, Arowojolu AO, Odukogbe AA, Yalew AW. Adverse neonatal outcomes of adolescent pregnancy in  Northwest Ethiopia. PLoS One. 2019;14(6):e0218259. | | Different outcome | Fikadu Wake | March 2024 |
|  | Gebrekidan MG, Fisseha MA, Gebreyesus A, Fisseha G. Effect of bleeding during Pregnancy on Maternal and  Perinatal Adverse Outcomes in Northern Ethiopia:-Using two years data from Hospital. | | preprint | Alex Ayenew | May 2024 |
|  | Syoum FH, Abreha GF, Teklemichael DM, Chekole MK. Fetomaternal Outcomes and Associated Factors among  Mothers with Hypertensive Disorders of Pregnancy in Suhul Hospital, Northwest Tigray, Ethiopia. J Pregnancy.  2022 Nov 9;2022:6917009. doi: 10.1155/2022/6917009. PMID: 36406161; PMCID: PMC9668464. | | Different outcome | Adamu Shibabaw | March 2024 |
|  | Fantahun, Y., Mesfin, E., & Assefa, addisu. (2020). Perinatal and Maternal Outcome of Antepartum Hemorrhage in Addis Ababa University . Ethiopian Journal of Reproductive Health, 12(3), 8.  https://doi.org/10.69614/ejrh.v12i3.395 | | Full text not accesible | Alex Ayenew | May 2024 |
|  | Lolaso T, Oljira L, Dessie Y, Gebremedhin M, Wakgari N. Adverse birth outcome and associated factors among  newborns delivered in public health institutions, Southern Ethiopia. East African Journal of Health and Biomedical Sciences. 2019 Nov 16;3(2):35-44. | | Different outcome | Adamu Shibabaw | March 2024 |
|  | Kebede AS, Muche AA, Alene AG. Factors associated with adverse pregnancy outcome in Debre Tabor town,  Northwest Ethiopia: a case control study. BMC Res Notes. 2018;11(1):820. | | Different outcome | Fikadu Wake | May 2024 |
|  | Abdo RA, Endalemaw TB, Tesso FY. Prevalence and associated factors of adverse birth outcomes among women attended maternity ward at Negest Elene Mohammed Memorial General Hospital in Hosanna Town, SNNPR, Ethiopia. J Women’s Health Care. 2016;5(4). | | Different outcome | Adamu Shibabaw | May 2024 |
