# Supplementary material for: Fetal outcomes and associated factors of antepartum hemorrhage in Ethiopia: A systematic review and meta-analysis
Source: PLoS One. 2025 Mar 4;20(3):e0319512. doi: 10.1371/journal.pone.0319512 (PMC11878924; doi:10.1371/journal.pone.0319512)
Supplement: S5 & 6 Table — (DOCX) [file pone.0319512.s005.docx]

**Supporting tables of all data extracted from the primary research sources for the systematic review and/or meta-analysis.**

**S5 Table**: Prevalence data extracted from the primary research sources for the systematic review and/or meta-analysis.

| **Authors** | **Year of study** | **Region** | **Study design** | **Study population** | **Sample Size** | **Response rate** | **Prevalence** | **logprevalence** | **seprevalence** | **selogprevalence** |
| --- | --- | --- | --- | --- | --- | --- | --- | --- | --- | --- |
| Asefa et al | 2020 | Addis Ababa | Cross-sectional | newborns | 9643 | 100.00% | 15.8 | 2.76001 | 10.52917 | 4.729194 |
| Hailu et al | 2023 | Tigray | Cross-sectional | newborns | 5368 | 100.00% | 22.5 | 3.113515 | 12.05456 | 5.013794 |
| Dasash et al | 2019 | Oromia | Cross-sectional | newborns | 3224 | 100.00% | 22.8 | 3.126761 | 12.11115 | 5.024104 |
| Zegeye et al | 2024 | Amhara | Cross-sectional | newborns | 448 | 100.00% | 17.4 | 2.85647 | 10.94395 | 4.808739 |
| Chufamo et al | 2015 | Oromia | Cross-sectional | newborns | 3854 | 100.00% | 30.6 | 3.421 | 13.30301 | 5.247196 |
| Gelan et al | 2022 | Oromia | Cross-sectional | newborns | 377 | 98.00% | 26.5 | 3.277145 | 12.74019 | 5.13951 |

**S6 Table: Variables data extracted from the primary research sources for the systematic review and/or meta-analysis.**

| **Authors** | **Associated factors** | **OR** | **LCI** | **UCI** | **logOR** | **logLCI** | **logUCI** | **seLogOR** |
| --- | --- | --- | --- | --- | --- | --- | --- | --- |
| Hailu et al | Rural residence | 2.75 | 1.24 | 6.09 | 1.011601 | 0.215111 | 1.806648 | 0.406004 |
| Dasash et al | Delay to seek care >12 hours | 14.5 | 1.2 | 17.56 | 2.674149 | 0.182322 | 2.865624 | 0.684516 |
| Zegeye et al | Rural residence | 1.7 | 1.09 | 2.66 | 0.530628 | 0.086178 | 0.978326 | 0.227589 |
| Zegeye et al | Delay to seek care >12 hours | 2.57 | 1.57 | 4.23 | 0.943906 | 0.451076 | 1.442202 | 0.252838 |
| Chufamo et al | Rural residence | 1.53 | 1.23 | 1.96 | 0.425268 | 0.207014 | 0.672944 | 0.11886 |
| Gelan et al | Prematurity | 9 | 5.92 | 16.52 | 2.197225 | 1.778336 | 2.804572 | 0.261795 |
| Gelan et al | Moderate vaginal bleeding before arrival | 7.04 | 2.14 | 23.1 | 1.951608 | 0.760806 | 3.139833 | 0.606895 |
| Gelan et al | Severe vaginal bleeding before arrival | 4.26 | 1.22 | 14.91 | 1.449269 | 0.198851 | 2.702032 | 0.638567 |
