# Supplementary material for: Fetal outcomes and associated factors of antepartum hemorrhage in Ethiopia: A systematic review and meta-analysis
Source: PLoS One. 2025 Mar 4;20(3):e0319512. doi: 10.1371/journal.pone.0319512 (PMC11878924; doi:10.1371/journal.pone.0319512)
Supplement: S7 Table — (DOCX) [file pone.0319512.s006.docx]

**S7 Table: Quality assessment of evidence-based intrapartum care practices and associated factors among obstetric care providers in Ethiopia.**

| References |  | **Theoretical approach** | | **Data collection** | **Study design** | **Validity** | | **Analysis** | | | | **Ethics** | | **Overall quality** | **Data extractor** | **Checker** | **Date** |
| --- | --- | --- | --- | --- | --- | --- | --- | --- | --- | --- | --- | --- | --- | --- | --- | --- | --- |
|  |  | Is a qualitative approach appropriate? | Is the study clear in what it seeks to do? | How well was the data collection carried out? | How defensible/rigorous is the research design/methodology | Is the context clearly described? | Were the methods reliable? | Are the data ‘rich’? | Is the analysis reliable? | Are the findings convincing? | Are the conclusions adequate? | Was the study approved by an ethics committee? | Is the role of the researcher clearly described? |  |  |  |  |
| Wodajo et al [18] | Yes | Appropriate | Clear | Adequately | Defensible | Clear | Reliable | Uncertain/not reported | Reliable | Convincing | Adequate | Yes | Yes | Medium | AAS | AAC | Oct. 15 |
| Sendekie et al [16] | 🗹 | Appropriate | Clear | Adequately | Defensible | Clear | Reliable | Rich | Reliable | Convincing | Adequate | Yes | not reported | Medium | GWK | AAC | Oct. 15 |
| Fisseha et al [27] | 🗹 | Appropriate | Unclear | adequately | Not defensible | Clear | Uncertain | Poor | Uncertain | Not convincing | Inadequate | Not reported | Not clear | low | GWK | AAS | Oct. 25 |
| Kasahun et al [19] | 🗹 | Appropriate | Clear | Adequately | Defensible | Clear | Reliable | Uncertain | Reliable | Convincing | Adequate | Yes | Uncertain | Medium | AAC | GWK | Oct. 27 |
| Mengistie et al [17] | 🗹 | Appropriate | Clear | Adequately | Defensible | Clear | Reliable | Rich | Reliable | Convincing | Adequate | Yes | Not clear | Medium | ATD | GWK | Oct. 17 |
| Tadesse et al [21] | 🗹 | Appropriate | Unclear | adequately | Defensible | Clear | Uncertain | Poor | Uncertain | Not convincing | Inadequate | Not reported | Not clear | low | AAC | AAS | Nov. 16 |
| Asmere et al [11] | 🗹 | Appropriate | Clear | Adequately | Not defensible | Clear | Reliable | Uncertain | Reliable | Convincing | Adequate | Yes | Uncertain | Medium | AAS | AWD | Oct. 22 |
| Kebede et al [28] | 🗹 | Appropriate | Clear | Adequately | Defensible | Clear | Reliable | Uncertain/not reported | Reliable | Convincing | Adequate | Yes | Yes | Medium | AAS | AWD | Nov. 02 |
| Yigzaw et al [25] |  | Appropriate | Clear | Adequately | Defensible | Clear | Reliable | Rich | Reliable | Convincing | Adequate | Yes | not reported | Medium | GWK | AAC | Nov 28 |
| Regasa et al [12] |  | Appropriate | Clear | Adequately | Defensible | Clear | Reliable | Rich | Reliable | Convincing | Adequate | Yes | Not clear | Medium | ATD | GWK | Dec. 01 |
| Dagne et al [20] |  | Appropriate | Clear | Adequately | Defensible | Clear | Reliable | Uncertain | Reliable | Convincing | Adequate | Yes | Uncertain | Medium | AAC | GWK | Nov 23 |
| Dewana et al [26] |  | Appropriate | Clear | Adequately | Defensible | Clear | Reliable | Uncertain | Reliable | Convincing | Adequate | Yes | Uncertain | Medium | AAC | GWK | Dec 12 |
| Silesh et al [24] |  | Appropriate | Unclear | adequately | Defensible | Clear | Uncertain | Poor | Uncertain | Not convincing | Inadequate | Not reported | Not clear | low | AWD | AAS | Nov 30 |

**Question codes:**

1. Was the sample frame appropriate to address the target population?

2. Were study participants sampled in an appropriate way?

3. Was the sample size adequate?

4. Were the study subjects and the setting described in detail?

5. Was the data analysis conducted with sufficient coverage of the identified sample?

6. Were valid methods used for the identification of the condition?

7. Was the condition measured in a standard, reliable way for all participants?

8. Was there appropriate statistical analysis?

9. was the response rate adequate, and if not, was the low response rate managed appropriately?
